# RNA-Seq Analysis Revealed circRNAs and Genes Associated with Abdominal Fat Deposition in Ducks

**DOI:** 10.3390/ani14020260

**Published:** 2024-01-14

**Authors:** Yunfeng Yang, Chunyan Yang, Zhong Zhuang, Jiaming Mao, Anqi Chen, Tingting Zhou, Hao Bai, Yong Jiang, Guobin Chang, Zhixiu Wang

**Affiliations:** 1 Key Laboratory for Animal Genetics & Molecular Breeding of Jiangsu Province, College of Animal Science and Technology, Yangzhou University, Yangzhou 225009, China; yangyf202101@163.com (Y.Y.); yangcy202109@163.com (C.Y.); zz150211@163.com (Z.Z.); 13952596198@163.com (J.M.); 13336295886@163.com (A.C.); ztt648715244@163.com (T.Z.); jiangyong@yzu.edu.cn (Y.J.); gbchang1975@yzu.edu.cn (G.C.); 2 Joint International Research Laboratory of Agriculture and Agri-Product Safety, The Ministry of Education of China, Institutes of Agricultural Science and Technology Development, Yangzhou University, Yangzhou 225009, China; bhowen1027@yzu.edu.cn

**Keywords:** RNA-seq, circRNA, ducks, fat deposition

## Abstract

**Simple Summary:**

Fat deposition affects the quality of meat ducks and their feed conversion efficiency. In this study, we analyzed the correlations between abdominal fat and other growth traits, and performed RNA-seq of the abdominal fat tissue of ducks with high and low abdominal fat ratio, exploring the key regulatory genes and circRNAs related to abdominal fat deposition. We investigated the functions of both source and target genes of these circRNAs and constructed a competing endogenous RNA network. The findings revealed several circRNAs and their relationships with microRNAs and messenger RNAs that are pivotal in duck abdominal fat deposition. The results of this study establish a groundwork for understanding the molecular mechanisms that regulate abdominal fat deposition in ducks, offering a theoretical reference for the selective breeding of high-quality meat-producing ducks.

**Abstract:**

Fat deposition is an important factor affecting meat quality and feed conversion efficiency in meat ducks. This study aims to identify key circRNAs and genes affecting abdominal fat deposition. The correlations between abdominal fat and other growth performances were analyzed in 304 F_2_ generation of Cherry Valley duck Runzhou Crested White ducks, and an RNA-seq analysis of abdominal fat tissues from ducks with high and low rates of abdominal fat was performed. Growth performance results showed that Abdominal fat ratio and Intramuscular fat were significantly higher in the high rates of abdominal fat (HF)group than in the low rates of abdominal fat (LF) group for ducks. RNA-seq analysis of abdominal fat tissue unveiled 85 upregulated and 72 downregulated circRNAs among the differentially expressed ones. Notably, 74 circRNAs displayed more than four-fold differential expression, constituting 47.13% of the differentially expressed genes. Functional enrichment analysis of the differentially expressed circRNA source and target genes indicated that 17 circRNAs might partake in regulating duck abdominal fat production by influencing pathways like PPAR signaling, lipid droplets, and triglyceride metabolism. Lastly, multiple circRNA-microRNA-messenger RNA interaction networks were constructed. The results of this study establish the groundwork for understanding the molecular mechanisms that regulate abdominal fat deposition in ducks, offering a theoretical reference for the selective breeding of high-quality meat-producing ducks.

## 1. Introduction

Meat ducks occupy an important position in poultry meat due to their short rearing cycle, fast growth rate and high meat yield [1]. Meat ducks have a high fat-deposition capacity. Large-scale and intensive farming increases the production of meat ducks but also deposits more fat in the abdomen [2]. Intramuscular fat affects meat flavor, pH, tenderness, and juiciness [3], while abdominal fat deposition negatively affects the taste of duck meat and reduces the feed conversion rate of ducks, thus affecting the economic efficiency of the farming industry [4]. Therefore, research has focused on effectively controlling abdominal fat deposition in ducks and determining the underlying genetic mechanisms. Abdominal fat deposition is a complex quantitative trait and an economically important trait in the duck meat industry. It is regulated by multiple factors, including transcription factors, functional genes, circular RNA (circRNA), and signaling pathways related to adipogenesis [5].

Circular RNAs (circRNAs), identified as a distinct non-coding RNA in the 1980s, are back-spliced from linear RNA, forming a closed ring structure through covalent bonds. They actively participate in regulating various biological processes [6]. Several studies highlight the involvement of circRNAs in regulating animal fat synthesis and metabolism [7]. For instance, *circOgdh*, located in the cytoplasm, acts as a sponge for *miR-34a-5p*, competitively binding to it and upregulating the expression of the key lipolysis gene, adipose triglyceride lipase (Atgl), enhancing the brown color of adipose tissue [8]. Lipolysis of adipocytes inhibits the accumulation of lipid droplets. In pigs, *circPPARA* adsorbs *miR-429* and *miR-200b*, promoting intramuscular adipogenesis [9]. Similarly, *chi-circ_0006511* binds to the novel R-87/fatty acid translocase (FAT)/*CD36* axis, positively regulating the differentiation of goat intramuscular adipocytes [10]. Studies on buffaloes reveal that *circMARK3* promotes the adipogenic differentiation of buffalo adipocytes and 3T3-L1 cells by upregulating the expression of the adipocyte marker genes *PPARG*, *C/EBPα*, and *FABP4* [11]. Compared to mammals, the understanding of duck circRNAs in adipogenesis is limited, necessitating detailed analyses to uncover the molecular mechanisms involved.

In recent years, RNA sequencing (RNA-seq) has been widely applied to construct circRNA expression profiles and explore their roles in avian fat proliferation and differentiation. For instance, Wang et al. screened 141 significantly differentially expressed circRNAs in duck adipocytes before and after an induction of differentiation [12]. Another study identified 41 significantly different circRNAs in chickens with varying abdominal fat levels. Subsequent bioinformatics analysis indicated that *novel_circ_PTPN2*, *novel_circ_TNNA1*, and *novel_circ_PTPRD* were involved in fatty acid metabolism, monocarboxylic acid metabolism, and carboxylic acid metabolism. Acid metabolism, glycerol metabolism process, fatty acid metabolism, and peroxisome proliferator-activated receptor (PPAR) signaling pathways are involved in chicken fat deposition [13].

This study utilized high-throughput RNA-seq to conduct an RNA-seq of abdominal fat in the F_2_ generation of Cherry Valley ducks × Runzhou crested white ducks with varying abdominal fat rates. Key circRNAs and target genes potentially regulating abdominal fat deposition are identified. The results of this study establish a groundwork for understanding the molecular mechanisms that regulate abdominal fat deposition in ducks, offering a theoretical reference for the selective breeding of high-quality meat-producing ducks.

## 2. Materials and Methods

### 2.1. Ethical Approval

The animal experiments performed in this study were reviewed and approved by the Animal Care and Use Committee of Yangzhou University (No. SYDW-2019015).

### 2.2. Test Animals and Tissue Samples

A total of 304 F_2_ generation of Cherry Valley duck ×Runzhou Crested White duck were selected from the Shuyang Zhongke Seed Poultry Co., Ltd. (Yangzhou, China). The ducks were fed and managed under the same environmental conditions with free access to feed and water and 24 h light. The temperature and humidity of the duck house were kept constant, so that the temperature was stable at 35 °C for the first week, and then gradually dropped to 20 °C. The humidity was kept above 60%. At 42 days of age, the ducks were euthanized, and measurements were taken for live weight, carcass weight, breast muscle weight, and abdominal fat weight. The moisture, protein, intramuscular fat (IMF), and collagen content of the meat were determined using near-infrared spectrophotometry [14].

Ducks were ranked according to abdominal fat rates and four duck abdominal fat samples were selected from the highest- and lowest-ranking groups as the high abdominal fat ratio group (HF) and low abdominal fat ratio group (LF), respectively, and named HF-1, HF-2, HF-3, HF-4, LF-1, LF-2, LF-3, and LF-4. Tissue samples were stored at −80 °C for backup.

### 2.3. RNA Isolation, Library Construction, Sequencing, and Data Quality Control

Total RNA was extracted from the abdominal fat tissue samples using TRIzol reagent (Invitrogen, Carlsbad, CA, USA). RNA concentration and quality were measured at OD 260/280 using the Nanodrop ND-2000 ultra-micro spectrophotometer (Thermo Fisher Scientific, Waltham, MA, USA). OD 260/280 in the range of 2.0–2.2 and RNA concentration around 500 ng/μL. The integrity of the RNA was measured by analyzing 2 µL of the total RNA on a 1% agarose gel. RNA libraries were then constructed using the TruSeq RNA-Seq Library Prep Kit v.2 (Illumina, Shanghai, China) following the manufacturer’s instructions. Subsequently, libraries were constructed and sequenced using a HiSeqTM 2000 instrument (Illumina), completed by Gene Denovo Biotechnology Co., Ltd. (Guangzhou, China).

To ensure high-quality clean reads, FASTP was used for quality control. The resulting high-quality clean reads were compared with a ribosomal database using Bowtie2 [15]. This comparison allowed for calculating the ratio of high-quality clean data to total RNA. The reads derived from the filtered ribosomes were aligned to the reference genome using TopHat2 [16]. Unmapped reads were extracted from the alignment results, and both ends of each unmapped read (default 20 bp) were intercepted to obtain the anchor reads. The anchor reads were then aligned to the reference genome.

### 2.4. Identification and Differential Expression Analysis of circRNAs

After aligning the anchor reads with the reference genome, the comparison results of all samples were combined, and the merged results were subjected to circRNA identification using the find_circ tool [17]. Subsequently, highly plausible circRNAs were identified by additional filtering. The identified circRNAs were subjected to statistical analyses to determine their types, chromosomal distributions, and lengths. This analysis integrated the information provided by Find_circ and the transcriptome annotation of the reference genome. For annotation, BLAST searches were performed for the identified circRNAs against the circBase database [18]. The circRNAs that could not be annotated were considered novel. The edgeR package (http://www.rproject.org/, accessed on 3 December 2022) was used to identify the differentially expressed circRNAs (DE circRNAs) across samples or groups. Among the high and low abdominal fat rate groups, circRNAs whose expression levels were associated with |log2(FC)| > 1 and *p* ≤ 0.05 were considered DE circRNAs.

### 2.5. Target Gene Prediction and Functional Enrichment Analysis of Differential circRNAs

For circRNAs annotated in circBase, the target relationship with microRNAs (miRNAs) was predicted using StarBase (v2.0) (https://rnasysu.com/encori/, accessed on 3 December 2022). For novel circRNAs, three software packages, Mireap (https://sourceforge.net/projects/mireap, accessed on 3 December 2022), Miranda (v3.3a) (http://www.microrna.org, accessed on 3 December 2022), and TargetScan (v7.0) (http://www.targetscan.org, accessed on 3 December 2022), were used to predict targets for animal samples, and Patmatch (v1.2) (ftp://ftp.arabidopsis.org/home/tair/Software/Patmatch/, accessed on 3 December 2022) was used to predict target genes for plant samples. miRTarBase (v6.1) (http://mirtarbase.mbc.nctu.edu.tw/php/index.php, accessed on 3 December 2022) was used to predict messenger RNAs (mRNAs) that interact with circRNAs and miRNAs. The resulting circRNA-miRNA-mRNA correlation was visualized using Cytoscape (v3.6.0) (http://www.cytoscape.org/, accessed on 3 December 2022). The source and target genes of the DE circRNAs were analyzed for Gene Ontology (GO) and Kyoto Encyclopedia of Genes and Genomes (KEGG) functional enrichment, leading to the identification of DE circRNAs associated with abdominal fat deposition.

### 2.6. qRT-PCR Validation of Candidate circRNAs

To validate the RNA-seq findings and ensure their reliability, qRT-PCR was performed to confirm the expression patterns of candidate circRNAs in the abdominal fat tissues of HF and LF ducks. Five circRNAs were randomly chosen, and primers for their amplification were designed. The details of these primers are listed in Table 1. To normalize the expression levels of selected circRNAs, *GAPDH* was utilized as the internal reference gene. The expression levels of the circRNAs were determined using the 2^−ΔΔCt^ method.

### 2.7. Statistical Analysis

One-way analysis of variance was conducted using SPSS 22.0 (IBM, Armonk, NY, USA). The significance between groups was determined using Dunnett’s method. Visualization of images was performed using GraphPad Prism software (version 8.0; GraphPad, San Diego, CA, USA). Each experiment was replicated at least three times, and the data are presented as mean ± standard error of the mean. A significance level of *p* < 0.05 was considered, while *p* < 0.01 was regarded as extremely significant.

## 3. Results

### 3.1. Abdominal Fat Ratio and Meat Quality Differences between HF and LF Groups

Effects of Abdominal fat ratio divergence on growth performance of ducks is shown in Table 2. Body weight (BW), Breast muscle ratio, Moisture ratio, Protein ratio and Collagen ratio of ducks at 42 days of age did not differ between the HF and LF groups (*p* > 0.05). Abdominal fat ratio was highly significant higher in the HF group than in the LF group, while Intramuscular fat ratio was significantly higher in the HF group than in the LF group (*p* < 0.01). The above shows that the intramuscular fat ratio correlates with the abdominal fat ratio.

### 3.2. Identification and Characterization of circRNA Library in Duck Fat Tissue

Utilizing circRNA sequencing analysis (Supplementary Table S1), we obtained the count of original reads from each library, ranging from 64,023,818 to 97,749,872 (Table 3). After eliminating unqualified reads, we assessed the quality of each library (refer to Table 3 and Table 4). Approximately 80% of the reads were effectively aligned with the reference genome (GCF_003850225.1), and the realignment rate of anchors with the reference genome ranged from 50% to 70% (Table 4). Moreover, using the Find_circ 2 (v1.99), we identified 7754 annotated circRNAs (Supplementary Table S2).

**Figure 1:**
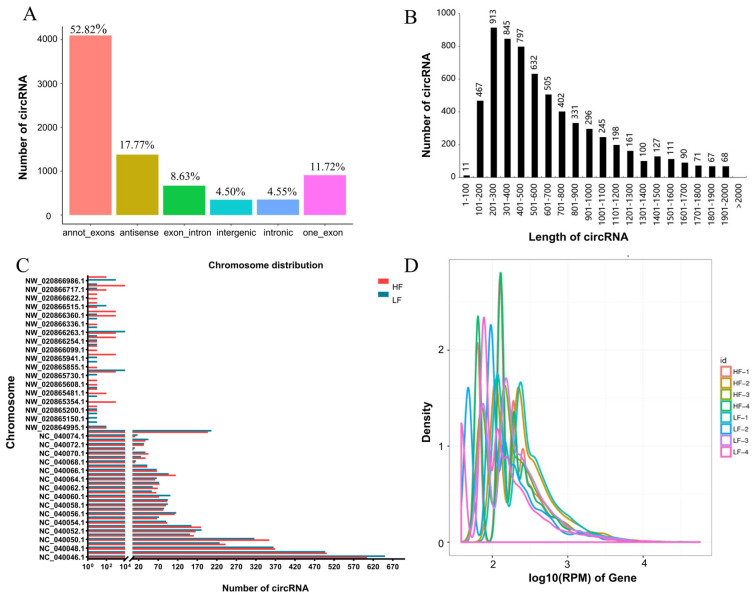
CircRNA identification statistics. (**A**) Numbers of identified circRNAs in various types; (**B**) Length distribution of all circRNAs; (**C**) Chromosome distribution of all circRNAs; (**D**) Expression abundance distribution map.

Among the six categorized circRNAs, annot_exons were the most prevalent, constituting 52.82% of the total circRNAs identified. Following this, antisense, one-exon, and intron-exons accounted for average percentages of 17.77%, 11.72%, and 8.63%, respectively. Intronic and intergenic sequences were the least common, with average proportions of 4.55% and 4.50%, respectively (Figure 1A). CircRNAs were distributed across most duck chromosomes, with the majority having lengths between 100 and 1000 base pairs (Figure 1B,C). The expression level test results indicated variations in transcript expression levels between the groups, and the trend in circRNA expression was similar (Figure 1D).

### 3.3. Analysis and Validation of Differentially Expressed circRNAs between Abdominal Fat of HF and LF Ducks

**Figure 2:**
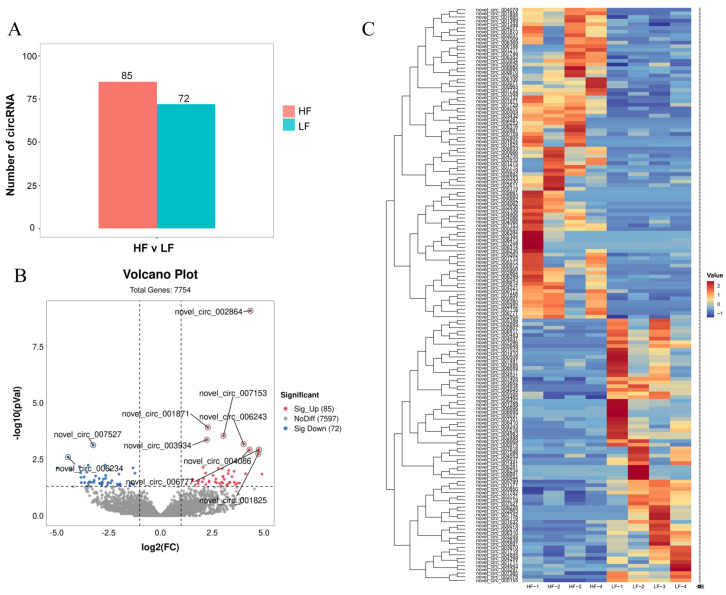
CircRNA differential expression statistics. (**A**) Statistics on the number of DE circRNAs in the high and low abdominal fat rate groups; (**B**) Volcano map of DE circRNAs, the Y axis is the value of −log10 (*p* Value). The X axis is the value of log2(FC). and the two threshold lines, respectively represent *p* = 0.01 and |log2(FC)|; (**C**) Heat map of DE circRNAs between in the high and low abdominal fat rate groups.

A total of 7754 circRNAs were identified in the abdominal fat tissue of ducks, with 157 circRNAs showing differential expression between HF and LF ducks (Supplementary Table S3). Among these, 85 circRNAs were upregulated, while 72 were downregulated in the abdominal fat tissues of HF ducks compared to LF ducks (Figure 2A,B). The heatmap demonstrated consistent differential circRNA expression within the groups (Figure 2C). The top upregulated circRNAs in HF ducks included *novel_circ_006284*, *novel_circ_006473*, *novel_circ_002344*, *novel_circ_000033*, and *novel_circ_007248*. Conversely, the most downregulated circRNAs in HF ducks were *novel_circ_006402*, *novel_circ_001688*, *novel_circ_005037*, *novel_circ_007351*, and *novel_circ_006410*. Among the differentially expressed circRNAs, 34 were exclusively expressed in HF ducks, and 28 were exclusive to LF ducks (Supplementary Table S3).

**Figure 3:**
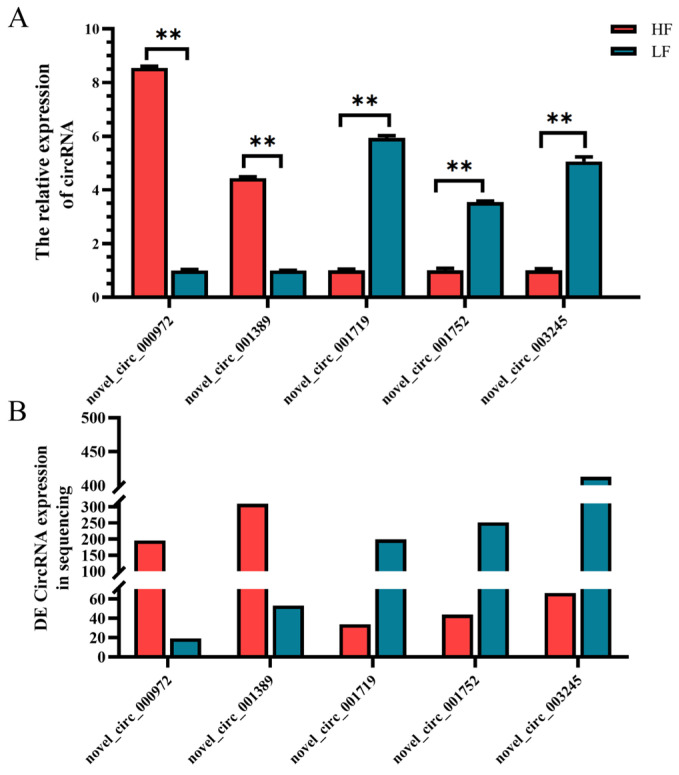
qRT-PCR verification of differentially expressed circRNAs result. Red represents HF and blue represents LF (** *p* < 0.01). (**A**) qRT-PCR showed DE circRNAs expression between the two groups; (**B**) sequencing results showed de expression between the two groups.

To further validate the RNA-seq data, five DE circRNAs were selected for reverse transcription-quantitative polymerase chain reaction (RT-qPCR) analysis. As depicted in Figure 3, the relative expression levels of *novel_circ_000972* and *novel_circ_001389* were higher in the abdominal fat tissues of HF ducks compared to LF ducks. Conversely, the relative expression levels of *novel_circ_001719*, *novel_circ_001752* and *novel_circ_003245* were lower in the abdominal fat tissues of HF ducks than in LF ducks (Figure 3). The RT-qPCR results corroborated that the relative expression levels of the DE circRNAs were consistent with those obtained from RNA-seq, confirming the reliability and repeatability of the RNA-seq results.

### 3.4. Functional Enrichment Analysis of DE circRNAs Source Genes

**Figure 4:**
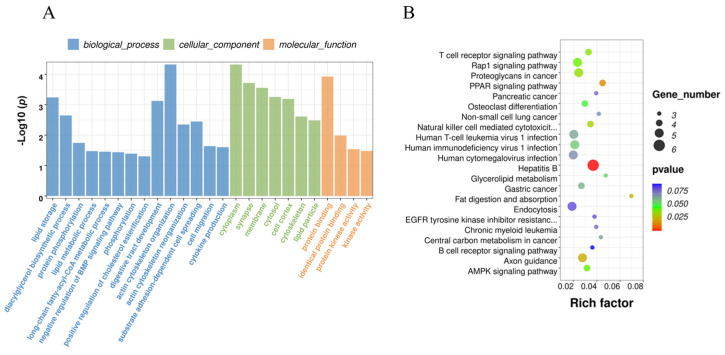
Analysis of Gene Ontology and KEGG Pathway Enrichment of source genes. (**A**) Gene Ontology of source genes. Blue column represents BP, green column represents CC and orange column represents MF; (**B**) KEGG Pathway Enrichment of source genes.

To further analyze the function of DE circRNA, GO and KEGG functional enrichment analyses were performed on all DE circRNA source genes. The source genes corresponding to the 157 DE circRNAs are listed in Supplementary Table S3. These source genes were enriched in 130 GO terms, of which 85 were significantly enriched (Supplementary Table S4). The results showed that the source genes of DE circRNAs were significantly enriched in guanyl-nucleotide exchange factor activity (*p* = 4.37 × 10^−5^), actin cytoskeleton organization (*p* = 4.75 × 10^−5^), cytoplasm (*p* = 4.77 × 10^−5^), neuromuscular junction development (*p* = 4.77 × 10^−5^), and protein binding (*p* = 8.07 × 10^−4^). The source genes of DE circRNAs related to lipid metabolism were significantly enriched in lipid binding (*p* = 0.0486), lipid storage(*p* < 0.01), lipid metabolic process (*p* = 0.0337) and lipid particles (*p* = 0.00327; Figure 4A). The source genes of DE circRNAs related to lipid metabolism were significantly enriched in pathways including AMPK signaling (*p* = 0.0442), fat digestion and absorption (*p* = 0.0291), and PPAR signaling (*p* = 0.0178; Figure 4B).

### 3.5. Functional Enrichment of DE circRNA Target Genes Related to Fat Deposition

**Figure 5:**
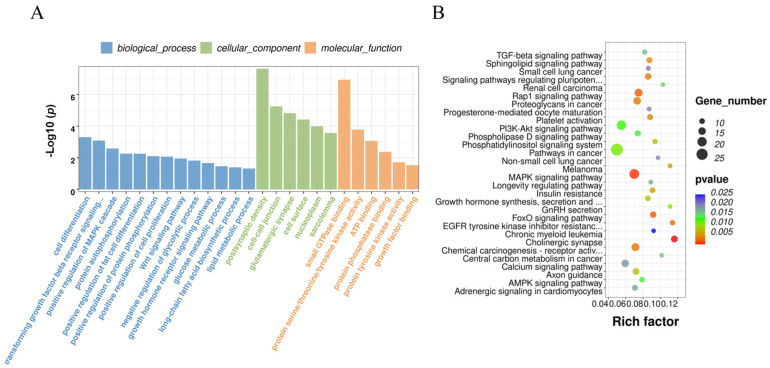
Analysis of Gene Ontology and KEGG Pathway Enrichment of target genes. (**A**) Gene Ontology of target genes. Blue column represents BP, green column represents CC and orange column represents MF; (**B**) KEGG Pathway Enrichment of target genes.

The DE circRNAs before and after reprogramming included 14 sponged miRNAs. The miRanda database was used to predict these miRNAs, and 856 predicted mRNAs were identified (Supplementary Table S5). GO and KEGG enrichment of target genes were performed for all DE circRNAs. GO and KEGG analyses were conducted on the target genes, revealing 437 GO entries and 68 KEGG pathways that were enriched (Supplementary Table S6). Target genes were found to be enriched in GO entries related to cell differentiation (*p* = 4.93 × 10^−4^), lipid metabolic process (*p* = 0.0483), long-chain fatty acid biosynthetic process (*p* = 0.0403), and glucose metabolic process (*p* = 0.0346), suggesting an association with lipid metabolism (Figure 5A). KEGG analysis showed that these genes were associated with adipogenesis-related signaling pathways and hormones, such as the MAPK signaling pathway (*p* = 6.59 × 10^−4^), the FoxO signaling pathway (*p* = 0.0018), the AMPK signaling pathway (*p* = 0.0135), the TGF-beta signaling pathway (*p* = 0.0168), insulin resistance (*p* = 0.0055), and GnRH secretion (*p* = 0.0109; Figure 5B).

### 3.6. Establishment of a Lipid-Deposition-Related Competing Endogenous RNA Network

**Figure 6:**
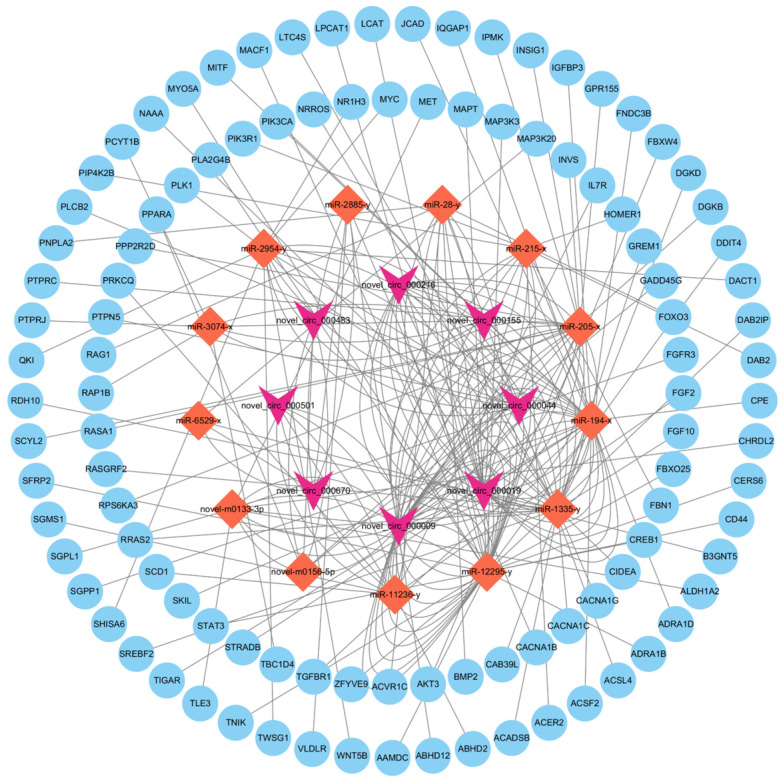
Analysis diagram of targeting relationships for DECs. circRNA-miRNA-gene targeting relationship, where inverted triangles represent circRNA, diamonds represent miRNAs, and circles represent genes.

To reveal potential interactions between circRNAs, miRNAs, and mRNAs in order to screen circRNAs and genes that can influence abdominal fat deposition. We identified and established a differential circRNA-miRNA-mRNA co-expression network. In total, 67 DE circRNAs, 14 miRNAs, 856 mRNAs, and 18,283 edges were identified in the ceRNA network, of which 37 were only miRNAs, respectively (Supplementary Table S7). Combined with the functional enrichment analysis of the source genes and target genes of DE circRNA, we found that 8 DE circRNAs regulated the expression of 105 genes related to lipid metabolism pathways by binding to 13 miRNAs (Figure 6). In our established ceRNA network, *novel_circ_000670*, with *miR-11236-y* adsorption, upregulated the target genes *SCD1*, *IL7R*, and *FGF2*, associated with lipid metabolism. With the adsorption of *miR-28-y*, *novel_circ_000019* upregulated *ACSL4* and *FOXO3*, linked to lipid metabolism. With the adsorption of *m0156-5p*, *novel_circ_000483* upregulated *PPARA*, associated with lipid metabolism. Therefore, we hypothesized that these circRNAs and these genes are critical in influencing abdominal fat deposition.

## 4. Discussion

Animal fat tissue predominantly consists of fat cells, which play a crucial role in metabolism by storing energy, exerting endocrine functions, and influencing animal health and meat quality [19]. Studies indicate excessive fat deposition escalates breeding costs and diminishes slaughter yields, financial returns, and consumer acceptance [20]. A prior investigation highlighted that for every 100 g increase in carcass weight, subcutaneous fat rises by 0.6%, accompanied by a 0.16% decline in muscle weight [21]. Our findings align, revealing the HF group’s higher body weight and lower chest muscle weight compared to the LF group. Notably, the HF group exhibited significantly higher IMF content. IMF, a prominent form of fat deposition, significantly impacts livestock and poultry meat quality [22]. Previous research establishes a robust correlation between IMF content and meat properties such as shear force, flavor, and tenderness. Increasing IMF content can enhance muscle tenderness, flavor, and juiciness [23]. Therefore, understanding the fat deposition mechanism becomes crucial for breeding to reduce fat deposition moderately, enhance lean meat production efficiency, and maintain excellent meat quality.

Recent advancements in non-coding RNA research, particularly circRNAs, have positioned them as novel regulatory factors in lipid metabolism [24]. Utilizing RNA-seq, we identified 7754 circRNAs in both HF and LF duck abdominal fat tissues, predominantly originating from exons. This aligns with prior studies indicating that exonic circRNAs are primarily cytoplasmic, hinting at their protein-coding potential [25]. The obtained data demonstrated high quality, with a 98.2% passing rate in quality control and 80% of reads successfully mapped to the reference genome, ensuring credibility in subsequent analyses. Notably, certain source genes of circRNAs exhibited a close association with fat metabolism. For instance, *SCD*, the source gene of *circ_004062*, plays a role in fatty acid composition and metabolism [26,27]. Additionally, an upregulated circRNA in the HF group, *circ_000033*, is derived from *DGAT2*, which catalyzes the covalent binding of diacylglycerol to fatty acyl-CoA to form triglycerides [28]. This process is crucial for integrating carbohydrate and fat metabolism in the liver [29]. Therefore, *circ_004062* and *circ_000033* potentially contribute to the disparity in abdominal fat deposition between the HF and LF groups. Subsequent GO and KEGG enrichment analyses on the source genes of circRNAs revealed significant enrichment in pathways closely associated with fat metabolism, such as the PPAR signaling pathway, fat digestion pathway, and glycerolipid metabolism. This implies that the DE circRNAs identified may influence duck fat deposition by modulating GO and KEGG pathways linked to their respective source genes.

Current research underscores the prominence of circRNAs as a prevalent form of non-coding RNA, characterized by wide distribution, high stability, and evolutionary conservation [30]. These molecules exert their regulatory functions by influencing transcription and mRNA turnover, playing a pivotal role in various biological processes, including fat synthesis and metabolism [31]. Notably, *circ-11103* has been shown to impact triglyceride levels and enhance unsaturated fatty acid abundance in bovine mammary epithelial cells by modulating the expression of *PPARGC1A* [32]. Similarly, the suppression of *circ-PLXNA1* expression in duck adipocytes leads to a significant reduction in the number of adipocytes [12]. To further explore the regulatory potential of circRNAs in fat synthesis and metabolism, we conducted a prediction analysis, identifying 856 candidate circRNA-targeted genes. Of these, 105 genes were enriched in pathways associated with fat metabolism, including lipid particles, the FOXO signaling pathway, the apelin signaling pathway, and glycerophospholipid metabolism. This suggests a significant involvement of the identified circRNAs in fat synthesis and metabolism.

circRNAs predominantly function as molecular sponges for miRNAs [33], binding to miRNAs as a competitive endogenous RNA (ceRNAs) to regulate the expression levels of target genes. This regulatory mechanism has been extensively studied for its role in adipogenesis and differentiation. For example, *chi-circ_0006511* has been shown to regulate the expression of *CD36* by adsorbing *miR-87*, significantly reducing the differentiation of goat intramuscular adipocytes [10]. Additionally, the cytoplasmic exon of *circDOCK7* acts as an miRNA sponge, sequestering *gga-miR-301b-3p* from *ACSL1*, which increases *ACSL1* mRNA levels after transcription, facilitating the proliferation and differentiation of adipocytes [34]. In this study, we constructed 18,283 circRNA-miRNA-mRNA targeting relationships involving 67 circRNAs, 14 miRNAs, and 856 mRNAs.

Previous research has demonstrated that PPARα regulates various genes related to lipoprotein lipase (*LPL*), apolipoproteins (*APOA1*, *APOA2*, and *APOA5*), fatty acid transport and oxidation (*FABP1*, *FABP3*, *ACS*, *ACO*, *CPT1*, and *CPT2*), high-density lipoprotein metabolism (*PLTP*), and ketone synthesis (*HMGCS2*), all of which stimulate fatty acid catabolism [35]. Furthermore, FOXO proteins are involved in regulating energy metabolism processes, such as glucose and fat metabolism, by affecting the expression of genes, such as glucose synthase (phosphotransferase 6 phosphorylase) and phosphoketoacid decarboxylase 4 (*PDK4*), thereby influencing the metabolic processes of glucose and fat [36]. Based on the findings of this study, we speculate that the *novel_circ_000483-novel-m0156-5p-PPARA*, *novel_circ_000019-miR-28-y-FOXO3*, and *novel_circ_000501-miR-11236-y-SCD1* axes may play crucial roles in the regulation of abdominal fat synthesis and metabolism in ducks.

## 5. Conclusions

In summary, growth performance results show that the intramuscular fat ratio correlates with the abdominal fat ratio. RNA-seq was used to study the abdominal fat tissue of four ducks in high and low abdominal fat rate groups. The analysis revealed 157 circRNAs that were differentially expressed between the two groups. By functional enrichment analysis of the source and target genes of DE circRNAs, five circRNAs (*circ_004062*, *circ_000033, circ_000483, circ_000019 and circ_000501*) were found to be involved in the regulation of abdominal fat deposition. And these five circRNAs target the expression of genes *SCD1, DGAT2, EVA1C, PGM2L1*, and so on. Therefore, it is hypothesized that these genes and circRNAs are involved in the regulation of abdominal lipid deposition. The results of this study establish the groundwork for understanding the molecular mechanisms that regulate abdominal fat deposition in ducks, offering a theoretical reference for the selective breeding of high-quality meat-producing ducks.

## Figures and Tables

**Table 1:** Primers for key candidate circRNAs.

circRNAs	Source Gene	Primer Sequences (5′→3′)	Product Size/bp
Reference gene	*GAPDH*	F:GGTTGTCTCCTGCGACTTCA R:TCCTTGGATGCCATGTGGAC	116
*novel_circ_00* *0972*	*DOCK4*	F: AATGCAGGTCTGGCAGTTTCT R: TCTGGACATTTTGGCAAACCAT	184
*novel_circ_00* *1389*	*VIM*	F: CATCTACGCCACCATGAGCA R: TGCTGCTCACCACGTAGC	104
*novel_circ_00* *1719*	*TRIO*	F: CCAGCGCCAACTGGAAAAC R:ACATACACATTCTGTTGAACATCCT	102
*novel_circ_001* *752*	*COBL*	F: GCTGGTCAATGGCTACATGG R: AGCGAAACACTTCGACTTGG	136
*novel_circ_00* *3245*	*MAEA*	F: CAAAGTGGTATTGAGGTGCCAT R: TCTGCCACCACCATCGTAAC	104

**Table 2:** Effects of Abdominal fat ratio divergence on growth performance of ducks.

Items	HF	LF	SEM	*p* Value
42 d BW (g)	2415.40	2166.20	140.53	0.09
Breast muscle ratio (%)	8.22	8.70	0.52	0.36
Abdominal fat ratio (%)	1.68	0.49	0.03	<0.001
Intramuscular fat ratio (%)	1.54	1.25	0.11	0.01
Moisture ratio (%)	76.80	77.35	0.33	0.11
Protein ratio (%)	22.19	22.10	0.36	0.80
Collagen ratio(%)	1.64	1.63	0.12	0.94

**Table 3:** Comparative statistics of high-quality clean data and rRNA.

Sample	All Reads Number	Unmapped Ratio	Mapping Ratio
HF-1	87,142,012	89.92%	10.08%
HF-2	67,035,628	75.00%	25.00%
HF-3	79,462,332	85.29%	14.71%
HF-4	87,183,872	76.30%	23.70%
LF-1	64,023,818	71.00%	29.00%
LF-2	97,749,872	96.48%	3.52%
LF-3	84,850,608	84.99%	15.01%
LF-4	89,618,340	91.50%	8.50%

**Table 4:** Screening of anchor reads and comparative statistics with reference genomes.

Sample	Total Reads	Mapping Ratio	Unmapped Ratio	Anchors Number	Mapped Anchors	Mapping Ratio
HF-1	78,360,250	83.08%	16.92%	26,513,884	16,595,685	62.59%
HF-2	50,275,220	80.52%	19.48%	19,586,420	10,861,523	55.45%
HF-3	67,770,884	83.08%	16.92%	22,933,808	13,822,768	60.27%
HF-4	66,524,018	83.13%	16.87%	22,450,874	13,451,724	59.92%
LF-1	45,459,676	79.80%	20.20%	18,362,120	9,580,656	52.18%
LF-2	94,305,132	83.36%	16.64%	31,391,256	20,007,383	63.74%
LF-3	72,114,628	84.30%	15.70%	22,648,508	14,544,476	64.22%
LF-4	82,004,742	85.06%	14.94%	24,508,692	16,125,034	65.79%

## Data Availability

All of the data generated or analyzed during this study are included in this published article.

## Supplementary Materials

The following supporting information can be downloaded at: https://www.mdpi.com/article/10.3390/ani14020260/s1, Table S1: Statistics for raw data quality control; Table S2: Statistical analysis of novel annotated circRNAs; Table S3: Statistical analysis of differential circRNAs; Table S4: Functional enrichment analysis of source genes; Table S5: Statistics for target gene prediction of differential circRNAs; Table S6: Targeting relationship between miRNAs and mRNAs for differential circRNAs; Table S7: Statistics for connectivity analysis scores of differential circRNAs.
